# Phase I Study of Anti-CD3 x Anti-Her2 Bispecific Antibody in Metastatic Castrate Resistant Prostate Cancer Patients

**DOI:** 10.1155/2015/285193

**Published:** 2015-02-23

**Authors:** Ulka Vaishampayan, Archana Thakur, Ritesh Rathore, Nicola Kouttab, Lawrence G. Lum

**Affiliations:** ^1^Department of Oncology, Wayne State University and Karmanos Cancer Institute, Detroit, MI 48201, USA; ^2^Department of Medicine, Wayne State University, Detroit, MI 48201, USA; ^3^Roger Williams Medical Center, Providence, RI 02908, USA; ^4^Department of Pathology, Roger Williams Medical Center, Providence, RI 02908, USA; ^5^Department of Immunology and Microbiology, Wayne State University, Detroit, MI 48201, USA

## Abstract

*Background*. New nontoxic targeted approaches are needed for patients with castrate resistant prostate cancer (CRPC). Our preclinical studies show that activated T cells (ATC) armed with anti-CD3 x anti-Her2 bispecific antibody (Her2Bi) kill prostate cancer cells lines, induce a Th_1_ cytokine pattern upon engagement of tumor cells, prevent the development of prostate tumors, and retard tumor growth in immunodeficient mice. These studies provided strong rationale for our phase I dose-escalation pilot study to test ATC armed with Her2Bi (aATC) for safety in men with CRPC. *Methods*. Seven of 8 men with CRPC were evaluable after receiving two infusions per week for 4 weeks. The men received 2.5, 5 or 10 × 10^9^ aATC per infusion with low dose interleukin-2 and granulocyte-macrophage colony stimulating factor. *Results*. There were no dose limiting toxicities, and there was 1 partial responder and 3 of 7 patients had significant decreases in their PSA levels and pain scores. Immune evaluations of peripheral blood mononuclear cells in 2 patients before and after immunotherapy showed increases in IFN-*γ* EliSpot responses and Th_1_ serum cytokines. *Conclusions*. These results provide a strong rationale for developing phase II trials to determine whether aATC are effective for treating CRPC.

## 1. Background

In 2010, the estimated mortality related to prostate cancer was noted to be increasing. With increasing numbers of prostate cancer patients with prostate specific antigen (PSA) relapse, the population with metastatic disease is expected to increase. Although androgen deprivation therapy (ADT) is initially very effective in patients, the disease eventually progresses into castrate resistant prostate cancer (CRPC). Failure of ADT is inevitable with disease progression associated with rising PSA levels, clinical symptoms, or abnormal scans. Despite treatment with docetaxel-based chemotherapy, patients with metastatic CRPC have an expected median survival of 18–20 months. Although targeted therapies such as abiraterone and MDV-3100 may improve overall survival, complete remissions are unlikely and patients eventually progress with pain, neuropathic compression, and fractures. Immunotherapy is an attractive nontoxic clinical strategy for patients with CRPC with the possibility of inducing prolonged remissions. Dendritic cell vaccine, Sipuleucel-T, has been shown to improve overall survival in patients with CRPC [1]. In 2010, Sipuleucel-T was approved by the US Food and Drug Administration (FDA) for patients with metastatic CRPC as an approved therapeutic option. The approval of Sipuleucel-T sets the stage for other cell-based immunotherapies for prostate cancer.

Her2/*neu* (Her2) overexpression as tested by immunohistochemistry (IHC) is reported in 42–70% of prostate cancers [2, 3]. Overexpression of Her2 appears to be an unfavorable prognostic factor and is associated with lower survival and higher relapse rates than those of Her2 negative prostate cancers [2]. Furthermore, serum Her2 levels were significantly higher in prostate cancer patients than in control subjects without cancer and serum Her2 levels were significantly higher in patients with metastatic disease than those without metastatic disease [4]. Likewise, in patients with metastatic disease, patients with higher serum Her2 levels had a shorter time to recurrence when compared to those with lower Her2 levels [4]. Her2 overexpression in prostate cancer cells also increased with progression to androgen independence [5, 6]. Forced Her2 overexpression in a LAPC-4 prostate cancer model confers androgen-independent growth to androgen-dependent prostate cancer cells [7]. These studies provide insights into the clinical role of Her2 expression in clinical progression and survival of patients with CRPC.

Several phase II clinical trials involved targeting Her2 prostate cancers by infusing trastuzumab (Herceptin) into prostate cancer patients; however, one study closed due to inability to accrue sufficient numbers of HER2+ patients [8] and another study failed to demonstrate benefit in CRPC patients [9]. The advantage of using anti-CD3 activated T cells (ATC) redirected by bispecific antibodies (BiAbs) over using monoclonal antibodies or BiAbs alone is that the arming of ATC with BiAbs combines independent mechanisms of cytotoxicity into a single biologic drug. Arming ATC with anti-CD3 x anti-Her2 BiAb (Her2Bi) targets T cells to Her2 on the tumor cells. Arming with Her2Bi transforms every ATC into a specific cytotoxic T cell directed at both high and low Her2 expressing targets. Our preclinical studies show that ATC armed with Her2Bi exhibited high levels of non-MHC restricted cytotoxicity directed at PC-3, DU-145, and LNCaP prostate cancer cell lines and produced tumoricidal cytokines such as interferon *γ* (IFN*γ*), tumor necrosis factor *α* (TNF*α*), and GM-CSF as well as MIP1alpha and RANTES [10–22]. Furthermore, Her2Bi-armed ATC coinjected with PC-3 localized to PC-3 xenografts and prevented growth of tumors or induced remissions when injected intratumorally in severe combined immunodeficient beige mice [23]. To evaluate this strategy, we performed a phase I trial evaluation of Her2Bi armed ATC infusions with specific focus on a CRPC cohort to review safety and preliminary efficacy. Evaluation of immune responses in our phase I clinical trial in CRPC patients suggests that infusions of Her2Bi-armed ATC (aATC) were safe and induced antitumor responses. Our findings suggest that Her2Bi-armed ATC therapy may be an effective, nontoxic, tumor-specific treatment for Her2-positive CRPC.

## 2. Patients and Methods

The study was a phase I trial conducted at Roger Williams Hospital, Providence, Rhode Island. The study and consent were approved by the local institutional review board. The primary objective of the study was to perform a phase I dose-escalation using Her2Bi armed ATC in prostate cancer patients with 0–3+ Her2 positivity. The cohort of patients with metastatic CRPC were treated under an FDA approved IND sponsored by LGL.

### 2.1. Eligibility for Prostate Cancer Cohort

Castration resistant patients with pathologically documented adenocarcinoma of the prostate that progressed despite androgen suppression therapy irrespective of their HER-2/*neu* expression status were eligible. Progression was determined by at least one of the following: rising PSA, increase in measurable disease, or new areas of bone metastases. Patients were required to have measurable or evaluable disease and at least 4 weeks of rest after chemotherapy or radiation before enrollment into the protocol. Her2 staining was not performed since it was not standard of care. Concurrent radiation treatment was not permitted; however, local irradiation of metastatic disease was allowed for local pain control and/or functional impairment due to localized lesions.

Cell infusions could begin as early as 1 week after completion of local irradiation if the toxicity had resolved based on the assessment of the treating physician. Karnofsky performance score ≥ 60% or ECOG score 0–2 was required, with minimum life expectancy of 3 months. Hormone therapy (except LHRH analogue) had to be discontinued at least four weeks prior to the initiation of armed-ATC infusions. Each patient had to sign a written informed consent to treatment after being informed of alternatives, potential benefits, side effects, and risks. No history of other malignancies was permitted unless it was* in situ* squamous cell carcinoma or basal cell carcinoma of the skin, or other cancers in remission for 5 years or more.

Exclusion criteria included history of myocardial infarction in last 12 months, impaired left ventricular function (LVEF ≤ 45% by MUGA), congestive heart failure, uncontrolled hypertension, or significant pulmonary disease (DLCO >60%) on pulmonary function tests. Normal bone marrow and renal and liver function were required. Patients with conditions or medications leading to immunosuppression were excluded.

### 2.2. Production of Heteroconjugated Bispecific Antibody (Her2Bi)

Anti-CD3 monoclonal antibody (OKT3, Centocor Ortho-Biotech, Raritan, NJ) was heteroconjugated to anti-Her2 monoclonal antibody (Herceptin, Genentech, South San Francisco, CA) to produce the anti-OKT3 x anti-Her2 bispecific antibody (Her2Bi) as previously described [24].

### 2.3. Leukopheresis, T Cell Expansion, and Arming with Her2Bi

peripheral blood mononuclear cells (PBMC) were collected to obtain lymphocytes for ATC expansion from 1 or 2 leukopheresis. PBMC were activated with 20 ng/mL of OKT3 and expanded in 100 IU/mL of IL-2 to generate 40–320 billion ATC during a maximum of 14 days of culture under cGMP conditions as described [15, 16]. The cells were grown in breathable flasks (FEP Bag Type 750-C1, American Fluoroseal Corporation, Gaithersburg, MD) in RPMI 1640 medium (Lonza) supplemented with 2% pooled heat inactivated human serum (Valley Biomedical, Winchester, VA). ATC were split approximately every 2-3 days based on cell counts. After 14 days of culture, ATC were harvested and armed with a pretitrated dose of 50 ng of Her2Bi/10^6^ ATC, washed, and cryopreserved in multiple aliquots. Aliquots of each bag were sent for bacterial and fungal cultures (Roger Williams Medical Center Pathology Laboratories), endotoxin testing (Lonza, Inc., Walkersville MD), and mycoplasma testing (Bionique Testing Laboratories, Inc., Saranac Lake, NY). No exogenous IL-2, OKT3, or other culture reagents (e.g., medium components) are present in the final cryopreserved product. The phenotype viability, proliferation, and responses of ATC to IL-2 did not change after cryopreservation. The cytotoxicity mediated by ATC armed prior to cryopreservation was equivalent to those armed after thawing of cryopreserved ATC.

### 2.4. Infusions of Her2Bi Armed ATC

**Figure 1:**
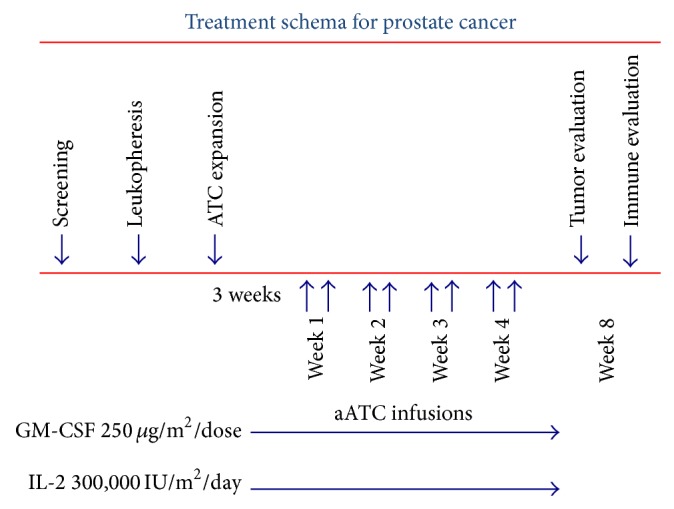
The treatment schema. Her2Bi armed ATC (aATC) were administered twice weekly for four consecutive weeks. All patients received subcutaneous IL-2 (300,000 IU/m^2^/day) and GM-CSF (250 *μ*g/m^2^/twice weekly), beginning 3 days before the first aATC infusion and ending 1 week after the last aATC infusion. Immune testing was performed at indicated time points.

T cells were infused only after standard operating procedures for identifying the cryopreserved product and the patient at the bedside. The infusion rate was calculated based on an infusing rate of no more than 5 EU of endotoxin/kg/hour. Armed ATC were given over 5–15 mins with monitoring of vital signs before and every 15 mins up to 1 hr after infusion. All vital signs and side effects were recorded on the patient's chart using the NCI immunotherapy toxicity table. Patients were observed up to 6 hours after their infusions. The dose levels for each infusion were 2.5, 5, 10, and 20 billion. Each patient would receive a total of 8 doses of armed ATC given twice weekly for 4 weeks. If the patients encountered toxicities related to armed ATC, modifications of the dose or holding the administration was required. The patients also received subcutaneous injections of interleukin-2 [IL-2] (3.0 × 10^5^ IU/m^2^/day) starting 3 days before the 1st armed ATC infusion and ending 7 days after the last armed ATC infusion. GM-CSF (250 *μ*g/m^2^ twice per week) was administered starting 3 days before the first armed ATC infusion and ending 7 days after the last dose of armed ATC (Figure 1).

### 2.5. Study Design and Statistical Plan

Three patients were entered at each dose level (Figure 1). Escalation of the dose was based on “cell-based” toxicity defined as toxicities attributed directly to the infusion of armed ATC. Dose escalation was done if more than 80% of planned-armed ATC dose was delivered without any grade 3 or greater toxicity attributed to therapy. The cohort was expanded to six patients at any particular dose level if one of the first three patients had their infusions stopped due to grade 3 or greater nonhematological toxicity or received less than 80% of planned dose of armed ATC. If, in the expanded cohort, one additional patient at this dose level had the infusions discontinued due to toxicities or had less than 80% of the planned dose delivered, then this particular dose level was to be defined as the one at which dose limiting toxicity (DLT) had occurred and further dose escalation was terminated. The maximum tolerated dose (MTD) would be defined as the dose level below the one at which dose limiting toxicity occurred and this would be the recommended phase II dose. The phase I trial consisted of metastatic breast cancer and prostate cancer patients. This is a report only on the prostate cancer patients enrolled on this study. Response was assessed by monitoring PSA every 4 weeks until progression. Due to small sample size, only descriptive clinical parameters of PSA decline, time to progression, and overall survival are used. Immunologic markers were assessed on the patients when cell numbers were permitted.

### 2.6. Immunologic Monitoring

The number of IFN*γ* producing cells from fresh PBMC from men before and at the various time points after infusions of armed ATC was detected by IFN*γ* specific EliSpot assay (BD Biosciences, San Jose, CA). Spots were captured and counted on CTL Immunospot counter using Immunospot software version 4 (Cellular Technology Ltd, Shaker Heights, OH). We compared whether* in vivo* lysis of tumors by the armed ATC will induce memory T cells capable of inducing apoptosis when exposed* in vitro* to tumor cells (a recall response) and to look for differences in the precursor frequency [25–29]. Mean changes in the induction of IFN*γ* EliSpots responses before and after immunotherapy were plotted over time to explore how long they can remain elevated. Paired *t*-test or Wilcoxon signed rank test was used in these comparisons. The means, medians, and standard deviations were calculated for the number of IFN*γ* EliSpots.

### 2.7. Endogenous Cytokine Production

In order to determine whether multiple infusions of armed ATC induced an immune response, Th_1_ and Th_2_ cytokines were evaluated in the serum of the patients using a 25-plex human cytokine Luminex Array (Invitrogen, Carlsbad, CA) on a Bio-Plex system (Bio-Rad Lab., Hercules, CA). Data was analyzed as function of number of infusions [25–29] and the* in vivo* response was calculated as the mean Th_1_[IL-2+IFN*γ*]/Th_2_ [IL-4+IL-5] ratio of cytokines.

## 3. Results

### 3.1. Patient Characteristics

Here we report pilot data on 8 CRPC patients from our phase I clinical trial; the remaining 22 metastatic breast cancer patients will be reported elsewhere. One of the enrolled CRPC patients did not receive therapy due to rapid progression of his disease. The median age of the 7 patients with metastatic CRPC treated on study was 76 years (range 66–85 years). Prior therapy included LHRH analogue therapy or antiandrogen therapy for all patients. One patient had received prior docetaxel-based chemotherapy, one patient had received prior mitoxantrone chemotherapy, and one patient had multiple hormonal maneuvers consisting of ketoconazole, steroids, and estrogen sequentially (Table 1).

### 3.2. ATC Characteristics

Patients received a total of 8 doses of 2.5, 5, 10, and 20 billion armed ATC given twice weekly for 4 weeks. Characteristics of ATC are shown in Table 2.

### 3.3. Clinical Responses, PSA Levels, and Toxicities

**Figure 2:**
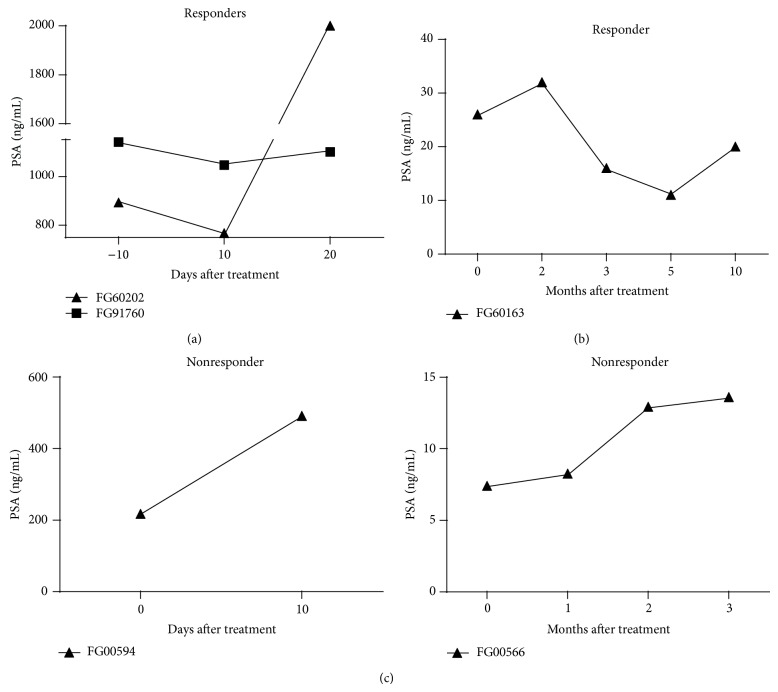
(a) The transient decrease in PSA levels in two CRPC patients (60202 and 91760) who had minor responses. (b) A partial responder (60163) with the PSA levels declining by >50% within 6 months of completing therapy. (c) PostIT PSA levels in two nonresponding patients (FG00594 and FG00566) compared to their preIT baseline levels.

There was a decrease in narcotic use in 2 of the 7 men possibly due to decreased bone pain. In addition to reduction of medication for bone pain associated with metastases, two CRPC patients with bulky disease infused with 40 (*60202*) and 80 (*91760*) billion Her2Bi-armed ATC, respectively, had minor responses observed as transient decreases in PSA levels (892 to 767 ng/mL and 1140 to 1046 ng/mL, resp.) that had short-term persistence after the last cell infusion (Figure 2(a)). A third CRPC patient (60163), who received 40 billion cells, had a* partial response* within 6 months of completing therapy with the patient's PSA levels decreasing by >50% (Figure 2(b)). Two patients showed an increase in their PSA levels after completion of 8 infusions of Her2Bi armed ATC compared to their preIT baseline levels (Figure 2(c)). Therapy was well tolerated and all patients received at least 80% of the planned dose. The doses administered, OS outcomes, and toxicities are included in Table 1. Our data show encouraging OS for a small cohort of men with HRPC.

### 3.4. Induction of Interferon Gamma (IFN*γ*) EliSpots

**Figure 3:**
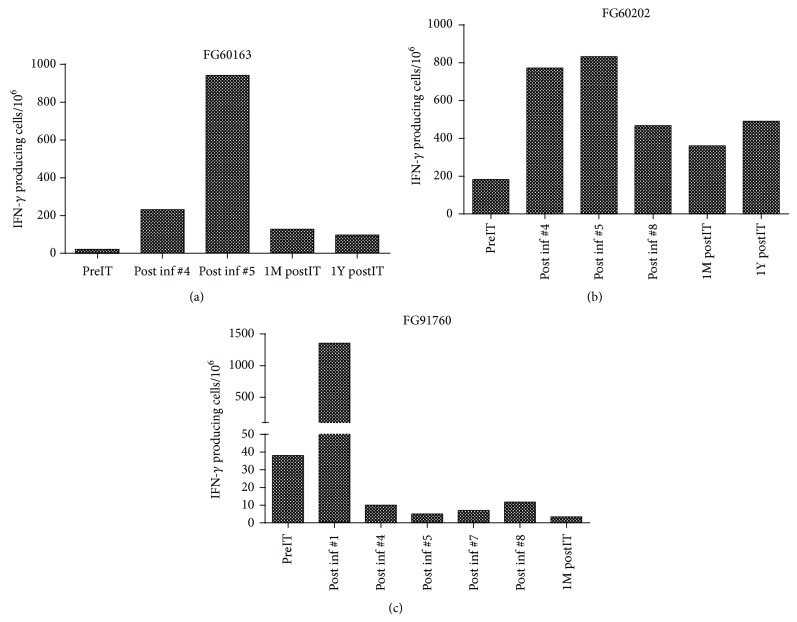
Number of IFN-*γ* producing cells when incubated overnight with prostate cancer cell line PC-3 in PBMC collected at baseline (preimmunotherapy [preIT]), during infusions (post inf#), and postimmunotherapy (1 week postimmunotherapy [1W postIT], 1 month postimmunotherapy [1 M postIT], and one year postimmunotherapy [1Y postIT]). (a) Increased number of IFN-*γ* producing cells during infusions and postIT time points in a partial responder (FG60163) when PBMC were incubated with prostate cancer specific PC-3 targets. (b) and (c) Enhanced IFN-*γ* responses in minor responders (FG60202 and FG91760).

Fresh PBMC were stimulated with PC-3 cells to induce IFN*γ* EliSpots. Figures 3(a), 3(b), and 3(c) show the increases in IFN*γ* expressing cells after armed ATC infusions. There was a noticeable increase in the number of IFN*γ* EliSpots at postinfusion #5 compared to baseline (prior to armed ATC infusions [preIT]) in two patients (FG60163 and FG60202) and postinfusion #1 in third patient (FG91760). These data show that either memory helper T cells or cytotoxic T cells were induced by Her2Bi armed ATC infusions, preexist in the circulating peripheral blood lymphocytes, and respond immediately to direct stimulation with prostate tumor antigens on PC-3 targets.

### 3.5. Infusions of Armed ATC Induce Endogenous Cytokine Production

**Figure 4:**
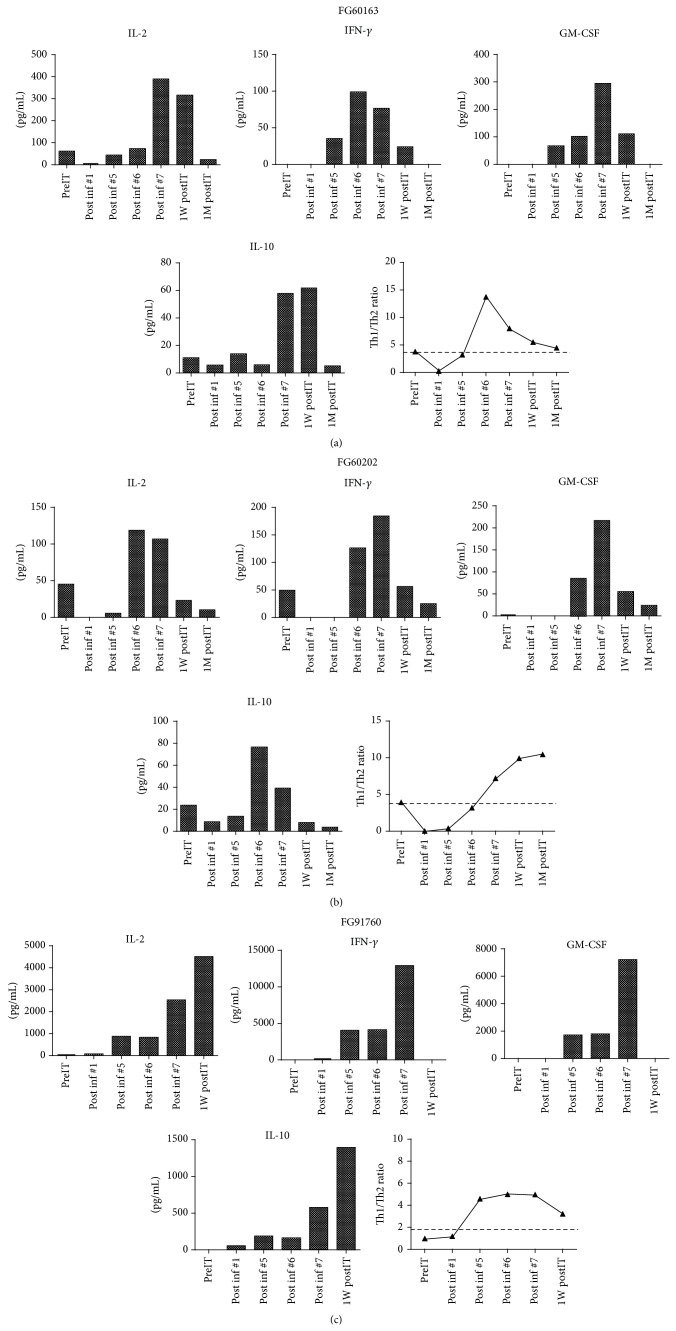
Profile of serum cytokines. Analysis of sequential serum samples at baseline (preimmunotherapy [preIT]), during infusions (post inf#), and postimmunotherapy (1 week postimmunotherapy [1W postIT], 1 month postimmunotherapy [1 M postIT], and one year postimmunotherapy [1Y postIT]) shows increased levels of IL-2, IFN-*γ*, GM-CSF, and IL-10 and meanratio of Th_1_/Th_2_ = [IL-2+IFN*γ*]/[IL-4+IL-10] shows a dominant Th_1_ type response during aATC infusions in partial responder CRPC patient (FG60163). (b) and (c) Similar cytokine profiles and Th_1_ cytokine responses were seen in two minor responder CRPC patients (FG60202 and FG91760) during and after aATC infusions.

The* in vivo* response calculated as the mean Th_1_[IL-2+IFN*γ*]/Th_2_ [IL-4+IL-5] ratio of cytokines remained predominantly Th_1_ polarized throughout treatment. Figures 4(a)–4(c) show Th_1_ and Th_2_ cytokines patterns for patients FG60163, FG60202, and FG91760, respectively. All three patients (2-MR and 1-PR) showed increases in IL-2, GM-CSF, and IFN-*γ* (Th_1_ cytokines) compared to baseline levels (preIT) in sequential testing of the serum. Th_2_ cytokine IL-10 also showed comparable pattern as Th_1_ cytokines. Other Th_2_ cytokines IL-4 and IL-5 could not be detected. These findings are consistent with increased specific cytotoxic T lymphocyte responses observed in EliSpots of patient postinfusion PBMC exposed to tumor cells. These observations show that the endogenous immune systems of cancer patients can be shifted to favor an antitumor immune environment by infusion of armed ATC.

## 4. Discussion

Our phase I study treating metastatic CRPC patients with Her2Bi-aATC shows that armed ATC infusions are safe and well tolerated leading to 2 minor and 1 partial responses in CRPC patients who received doses of 40 and 80 × 10^9^ aATC, respectively. The PSA levels decreased in 3 of 7 patients and one of the 7 patients had a >50% decline in PSA below the baseline (PR). In two patients who were studied for IFN-*γ* EliSpots and cytokine response, there was a marked increase in IFN-*γ* EliSpots and serum Th_1_ cytokines (IL-2, GM-CSF, and IFN-*γ*) after the 5th infusion (Inf #5) over baseline when no increases in Th_2_ cytokines were observed.

Armed ATC not only may function as Her2 specific cytotoxic T lymphocytes in prostate cancer patients, but also may divide and secrete Th_1_ cytokines and RANTES and MIP1-*α* after binding to Her2+ tumors leading to recruitment of endogenous T cells and monocytes leading to* in situ* vaccination at the tumor site [30]. Tumor lysis mediated by targeting T cells together with release of IL-2, IFN-*γ*, and GM-CSF may provide the milieu to induce immunization of endogenous immune cells to tumor associated antigens (TAA). Therefore, multiple rounds of armed ATC infusions could then eventually induce systemic immune responses that could be detected as IFN-*γ* EliSpots in the peripheral blood mononuclear cells (PBMC) from patients.

Recently, Sipuleucel-T/APC8015/Provenge showed a relapse free survival benefit in a phase III placebo controlled trial [1]. For this study, PBMC were collected and pulsed with prostatic acid phosphatase antigen and GM-CSF. The antigen loaded dendritic cell enriched preparation was then administered to patients. Final results of the placebo controlled trial involving 127 patients revealed an improvement in OS at 3 years. OS was 11% in the placebo group and OS was 33% in the Sipuleucel treated metastatic prostate cancer cohort (*P* = 0.02). The results of a larger phase III placebo controlled trial [IMPACT1] in asymptomatic metastatic CRPC patients showed an OS benefit with the therapy, but no PFS benefit. The agent was well tolerated and, due to the overall survival benefit, the FDA approved Sipuleucel-T as the first cell-based immunotherapy approved by the FDA for any cancer.

Other immunotherapy approaches have been tested in patients with metastatic CRPC. Initial studies with GVAX, an allogeneic cellular therapy consisting of a combination of two prostate carcinoma cell lines (PC-3 and LnCAP) with a modified GM-CSF gene, appeared to be promising. In phase II trials, GVAX was well tolerated and showed clinical promise with a median survival of 26 months and median time to progression of 5 months in patients with bone metastases [31]. However, a phase III trial of single agent GVAX versus docetaxel in asymptomatic CRPC was closed early due to a disappointing interim analysis, and a phase III trial of docetaxel +/− GVAX in symptomatic CRPC showed an increased number of deaths [24]. Recently, a PSA directed therapeutic vaccine (ProstVacVF) revealed a 44% reduction in death rate favoring the vaccine arm over placebo control after 13 years of follow-up [32]. There was no change in PFS when the primary endpoint of PFS was reported in 2004, but after prolonged follow-up, a statistically significant OS benefit was noted.

There is encouraging preliminary evidence to support investigations targeting CRPC with aATC. Multiple investigators have used infusions of BiAb to activate T cells* in vivo* and enhance targeting and killing of Her2^+^ tumors [33–38]. In a phase II advanced prostate and renal cancer trial using the BiAb MDX-H210 (15 *μ*g/m^2^) IV and GM-CSF 5 *μ*g/kg/day subcutaneously for 4 days repeated weekly for 6 weeks, 7 of 20 (35%) patients with prostate cancer had objective responses (>50% decline in PSA) [38].

There are a number of innovative and novel components of this approach. As described earlier, aATC can even target breast cancer cells lines [24] and prostate cancer cell lines (PC-3, LNCap) [23] with low HER2 receptor expression. The ability to target tumors that express low numbers of Her2 receptors is rationale for including all patients regardless of their Her2 expression. This study shows that multiple infusions of aATC induced immune responses that could be detected by reexposure to prostate cancer antigens in IFN-*γ* EliSpot assays. These data suggested that the armed ATC infusions “vaccinated” the patients against a whole array (unknown) of their own prostate cancer antigens and generated anti-tumor CTL or T cell helper activity. In an earlier study, we showed that in women with metastatic breast cancer low dose IL-2, low dose GM-CSF, and eight Her2Bi armed ATC infusions induced cytotoxic T cell activity directed at SK-BR-3 breast cancer cell targets that could be detected up to 4 months or more after immunotherapy [30]. The results from these studies suggest that this novel combination of antibody targeting and non-MHC restricted T cell-mediated cytotoxicity may have high clinical impact and therapeutic benefit in metastatic CRPC patients.

## 5. Conclusions

This phase I dose escalation study showed that the treatment of metastatic CRPC patients with Her2Bi-aATC was safe and well tolerated. CRPC patients who received 40 and 80 × 10^9^ aATC doses showed 1 partial and 2 minor responses, as well as induced antitumor immune responses. These data provide a strong rationale for developing phase II clinical trials to determine whether aATC are effective for treating CRPC.

## Figures and Tables

**Table 1:** Patient characteristics, prior therapy, disease status, and toxicity.

	FG #	Age/PS	Gleason score	aATC dose level	Metastatic disease sites	Prior therapy	Disease status	OS in days	Grade 3 toxicity
1	00469	66/PS-2	7	20 billion	Bone, lymph nodes, lung, and liver	Hormones docetaxel + estramustine	Progressed during aATC infusions	67	None

2	00488	82/PS-2	9	20 billion	Bone	Hormones mitoxantrone	Progressed during aATC infusions	143	None

3	00566	75/PS-1	8	20 billion	Bone	Hormones	Progressed during aATC infusions	699	Chills

4	00594	85/PS-1	8	20 billion	Lymph nodes, bone	Hormones	Progressed during aATC infusions	136	Chills

5	60163	76/PS-1	6	40 billion	Bone, lung	Hormones	PR	598	Chills

6	60202	80/PS-1	8	40 billion	Bone	Hormones	MR	294	Chills

7	91760	69/PS-1	6	80 billion	Bone, lung	Hormones	MR	550	Chills, Malaise

PS: performance status; PR: partial remission; MR: minor response; OS: overall survival. There were no grades for toxicities on the NCI-immunotherapy toxicity protocol.

**Table 2:** Phenotype of harvest product.

FG #	Total harvest ×10^9^	% viability	%CD3 harvest	%CD4 harvest	%CD8 harvest
1	35	95	89	58	34
2	38	95	93	75	22
3	48.6	91	99	50	50
4	65.4	87	79	19	60
5	139	94	89	61	35
6	171	87	90	78	16
